# The synergistic effect of carbon performance and technological innovation on corporate financial performance

**DOI:** 10.3389/fpsyg.2022.1060042

**Published:** 2022-12-06

**Authors:** Jin Yan, Hui Zhou, Jialin Mu, Ying Zhang, Airin Rahman

**Affiliations:** ^1^Business School, Hohai University, Nanjing, China; ^2^School of Finance and Economics, Jiangsu University, Zhenjiang, China; ^3^Department of Agribusiness and Marketing, Sher-e-Bangla Agricultural University, Dhaka, Bangladesh

**Keywords:** energy industry, carbon performance, technological innovation, corporate financial performance, low carbon economy

## Abstract

In order to further implement the goal of “dual carbon” proposed by China’s government, and promote energy enterprises to carry out low-carbon economic transformation, this paper takes listed companies in China’s A-share energy industry from 2014 to 2019 as samples to conduct descriptive statistics, correlation test and regression analysis, and empirically studies the impact of carbon performance and technological innovation on financial performance of China’s energy industry as well as their roles under different property rights. At the same time, the variables were delayed for one period to investigate the sustainability of carbon performance and technological innovation on financial performance and to weaken the endogeneity of the reverse causality between financial performance, carbon performance and technological innovation. The results show that good carbon performance and technological innovation in the energy industry can positively affect the financial performance of enterprises. During the research of interactive relationship, we find that carbon performance and technological innovation have synergistic effect on energy firm’s financial performance, which means technology innovation can significantly positive to adjust the action of carbon performance on financial performance,and carbon performance at the same time can also be positive to adjust the action of technology innovation on financial performance. They mutually promote energy enterprise’s financial performance. Further experimental research among different property- rights-owned enterprises, we found that the synergistic effects of carbon performance and technological innovation on corporate financial performance is much more significant in non-state-owned enterprises, possibly due to private firms’ capital profit-seeking nature. The results will guide and inspire China’s energy enterprises’ low carbon development strategy formulation and implementation under the background of “dual carbon” goal.

## Introduction

In recent decades, the climate problem has become one of the biggest problems in the society. Which in turn causes a lot of health problems (Wang et al., 2021). China will account for about half of the world’s carbon emissions in the next two decades as the global greenhouse effect continues to intensify due to increasing carbon emissions. Wesseh and Lin (2018). China has raised the “dual carbon” goal, which commits to reduce its carbon emissions by 40–45% by 2020 and by 60–65% by 2030 from the 2005 level. Specifically, our country has adopted a series of measures such as adjusting the industrial structure, optimizing the energy structure, saving energy and improving energy efficiency, promoting the construction of the carbon market, and increasing forest carbon sinks, etc., which have obtained remarkable achievements in tackling climate change. Obviously, the development of a low-carbon economy by enterprises has become an irresistible international trend. China’s carbon emissions are still among the highest in the world (Zhu et al., 2020b). China’s energy sector is the largest contributor to greenhouse gas emissions (Qamar Uz et al., 2021). However, as a key industry for our country to achieve carbon peaking and carbon neutrality goals, how can the energy and power industry realize a low-carbon transition under such “dual carbon” goal? According to the traditional concept, enterprises have born the extra cost of carbon emissions, which is not conducive to the improvement of enterprise performance (Shukla et al., 2008; Zhu et al., 2020a). Carbon emission taxes is not helpful to economic growing (Tu et al., 2022). So that they are reluctant to engage in carbon reduction activities. The efforts made by enterprises to reduce carbon emissions are not in vain. While reducing carbon dioxide emissions, they also improve corporate performance. For example, driven by technological innovation, combined with carbon emissions reduction measures, enterprises have produced a series of forward-looking green technologies while meeting the requirements of environmental regulations (Shamal et al., 2021), thus boosting production efficiency, reducing energy consumption and improving the competitiveness of enterprises. In the current era driven by innovative development, technological innovation is the fundamental guarantee for enterprises to obtain sustainable competitiveness, and the direction of innovation undoubtedly determines the fundamental benefits brought by itself. From the perspective of technical economy, it means to carry out “responsible investment.” The various carbon reduction measures undertaken by companies in the context of carbon reduction activities provide a new direction for technological innovation, allowing companies to develop a unique competitive advantage in line with the current environmental policy.

From the existing research, there are many studies on the relationship between corporate technological innovation and financial performance, most of which show a positive contribution, but there are also negative and unrelated explanations. Only few studies focus on carbon performance, and most of them stay in the aspect of social responsibility. However, Unlike carbon performance, which requires a large amount of cost input of enterprises, the performance of social responsibility is multi-faceted. It often has a certain disguise (He et al., 2017), which cannot well reflect the value of developing a low-carbon economy for enterprises. And how to balance economic development and environmental protection has always been one of the important issues that many scholars focus on. The innovation of this paper lies in the addition of technological innovation as a moderating variable. In addition to studying the impact of carbon performance and technological innovation on financial performance, it also tries to verify that both of them have moderating effects in the model, and they promote each other to improve the financial performance of enterprises. Based on this, the paper deeply studies the synergistic effect of corporate carbon performance and technological innovation on financial performance, and deeply explores the win-win situation of economy and environment under the “dual carbon” goal, providing theoretical support for our country’s commitment to reduce carbon emissions.

In this paper, hypotheses are first proposed through literature review, variables are then set up and models are constructed, and the relationship between carbon performance, technological innovation and financial performance is obtained through descriptive statistics, correlation analysis, regression analysis and robustness test. Finally, targeted suggestions are put forward accordingly.

Carbon performance is an important aspect of environmental performance, which shows the efforts and achievements of enterprises for carbon emission reduction. Technological innovation is generally manifested as R&D capital investment. The empirical results show that both carbon performance and technological innovation in the energy industry can significantly improve the financial performance of enterprises, and the two have synergistic effects to jointly improve the financial performance of enterprises. Further distinguishing the different property rights, in testing the impact of carbon performance and technological innovation on financial performance in state-owned enterprises and non-state-owned enterprises, we find that carbon performance is significantly positive in both samples, but technological innovation and interaction terms have no significant impact in state-owned enterprises. The findings from the study contribute to how to achieve the dual carbon goal and the reform of owned enterprise system. The results call for more investment in carbon reduction technology.

## Related literature and hypothesis development

### The impact of carbon performance on financial performance

From the existing research, the research on the environment first started from the concern of corporate social responsibility, and most of the studies show that the performance of corporate social responsibility has a significant role in promoting financial performance. Sayekti (2015) found that strategic corporate social responsibility is positively related to financial performance, while non-strategic corporate social responsibility is negatively related to financial performance. Carrying out carbon emissions reduction activities for enterprises is an important aspect of fulfilling their social responsibilities. The reduction of carbon emissions leads to the reduction of energy consumption (He et al., 2017), especially for energy companies, which can continue the capture of corporate value. Lu et al. (2021) found that the positive impact of carbon disclosure on financial performance can be extended to the next period. Therefore, formulating active and effective carbon strategies can not only adapt to today’s development trends, but also meet the needs of stakeholders. While there is little previous evidence that good corporate carbon performance can contribute to financial performance, or even scepticism about the contribution of carbon performance (Zhou et al., 2017). It is argued that corporate value can be devalued when companies implement carbon reduction activities that are inconsistent with the goal of profit maximization (Walley and Whitehead, 1994). However, on the one hand, based on the risk reward theory, carbon emissions reduction activities conducted by the enterprise reduce the risk of the government regulation and high penalty costs (Pearce, 1991). On the other hand, the reduction of carbon emissions means a reduction in energy consumption or an increase in productivity (Gaigné et al., 2020), which will send a positive signal to the outside world and make it easier to gain the attention of investors (Freedman and Jaggi, 1992), which will positively contribute to the development of the company. To sum up, companies with good carbon performance will bring the following positive effects: Firstly, the better the carbon performance of an enterprise, the more positive signals it sends to the outside world that its development is in line with the current economic situation, establishing a good corporate image that responds positively to the national policy and strengthening the confidence of investors. Secondly, good carbon performance indicates that the company has an irreducible advantage in terms of environmental cost, which improves the competitive advantage of the company’s products and sustainable development (Liu et al., 2022). Zhu and Zhang (2022) believe that energy enterprises focusing on environmental performance can optimize resource allocation and promote enterprise development. Liu and Zhao (2022) explored the relationship between carbon performance and financial performance based on the data of listed companies in China’s electricity and heat production and supply industries from 2012 to 2019, and found that carbon performance positively affected financial performance. Li and Lin (2021) took the data of 106 Chinese A-share listed companies from 2011 to 2018 as samples. Based on the study of the correlation between environmental performance and financial performance, they introduced investor confidence as A mediating variable to conduct multiple regression analysis to study its mediating effect on the relationship between the former two. The results show that improving environmental performance will improve financial performance. Yan et al. (2021) took A-share manufacturing listed companies in Shanghai and Shenzhen Stock exchanges from 2006 to 2016 as samples, estimated corporate carbon performance through industrial carbon emissions, and analyzed the impact of carbon performance on financial performance. The results show that the improvement of carbon performance is conducive to the increase of financial performance, and the responsibility of emission reduction can optimize the image of enterprises and improve the relationship with stakeholders.

It can be seen that good carbon performance behaviors by energy companies can effectively reduce energy costs, fulfill environmental responsibilities, and highlight image value among high-emission companies, which in turn makes them well reflected in the market and promotes the improvement of their financial performance. Based on this, we propose the hypothesis that:

*H1*: Carbon performance has a positive effect on corporate financial performance.

### The impact of technological innovation on financial performance

The current national development strategy proposes that scientific and technological innovation, which must be placed at the core of the overall national development, is the strategic support for improving social productivity and comprehensive national strength and that the adherence to the implementation of the innovation-driven development strategy should be emphasized, which identifies the important role of R&D innovation for economic development from the national level role (Yeguang and Bo, 2018). From the perspective of sustainable development of enterprises, the resource-based view doctrine proposes that technological innovation in enterprises is one of the most precious resources that can improve the core competitiveness and can bring differentiation of products or services, such as lower cost, higher quality or enable enterprises to gain competitive advantage in the market, and ultimately enhance enterprise value. At the same time, technological innovation brings unique resources to firms, which are often heterogeneous and difficult to replicate among firms, providing an irreplaceable advantage for firms’ competition (Yuan and Guangpei, 2021; Lei et al., 2022). According to the signaling theory, investors have more confidence and higher expected value for the future operation of enterprises with large investment in technological innovation. Lin et al. (2006) used a sample of U.S. firms and found that firms with more patents have better corporate performance. Therefore, under the internal efficiency improvement and the external good signal effect, more investment in technological innovation, especially when forming intangible assets, can directly improve the financial performance of enterprises (Hamamoto, 2006). Tariq et al. (2019) studied the impact of green product innovation performance on corporate financial performance based on 202 publicly listed Thai manufacturing enterprises, and the results showed that there was a significant positive relationship between them.

It can be seen that good technological innovation brings irreplaceable competitive advantages to the development of enterprises, especially for energy enterprises, whose main cost is energy consumption. Through reducing energy consumption and lowering the corresponding energy costs, technological innovation provides enterprises with a cost-competitive advantage. At the same time, technological innovation shows the development potential and motivation to external investors, which will further promote the development of the enterprise and thus improve the financial performance. Based on this, we propose the hypothesis that:

*H2*: Technological innovation has a positive effect on corporate financial performance.

### The impact of carbon performance and technological innovation on financial performance

For the development of an enterprise, it is necessary not only to pay attention to the interests of shareholders, but also to meet the requirements of stakeholders such as the government and society. Blindly innovating without a purpose often leads to counterproductive results, that is, a large number of inputs may lead to little output, which will not bring the expected economic benefits to the enterprise, but even bring a lot of losses to the enterprise.

Therefore, on the one hand, the choice of the direction of innovation is crucial. The background of implementing low-carbon strategies and actively carrying out corresponding measures provides a positive and effective direction for enterprise innovation, fulfilling the relevant social and legal responsibilities as well as meeting the requirements of national environmental control (Guan and Zhao, 2018), broadening financing channels, reducing financing costs, providing financial support for technological innovation, and forming green technology innovation (Heng and Yuhui, 2022). The reduction of energy consumption cost and pollution cost of the enterprise enables the enterprise to enhances product quality and have a lasting competitive advantage (Mensah et al., 2019; Zhang et al., 2020), so that it can better serve the development and improve the financial performance. On the other hand, technological innovation is one of the important ways for enterprises to fulfill their environmental responsibilities. In the context of technological innovation, the carbon emissions reduction activities of enterprises are not selfless investment, but those can be based on the focus of technological innovation to form green patented technologies, revealing to the outside world such a good signal that these activities are supported by corresponding technological innovation and have formulate a positive and effective carbon strategy. As a result, these activities can strengthen the determination of investors and dispel consumers’ purchase concerns, and promote the development of enterprises (Xiaobei and Xueting, 2021). Obviously, good technological innovation ability can help enterprises implement carbon strategy, thus improving the financial performance (Fisher and van Marrewijk, 1998; Takeda and Arimura, 2021).

In summary, good carbon performance behavior provides direction for technological innovation of enterprises, and technological innovation capability also provides guarantee to implement carbon strategy. The two promote each other and improve the financial performance, which means carbon performance can significantly positive to adjust the action of technology innovation on financial performance and technology innovation at the same time can also be positive to adjust the action of carbon performance on financial performance. They work together to promote the improvement of corporate financial performance. In addition, as there are two different types of enterprises in China, non-state-owned enterprises are more profit-seeking and have to maximize their profit in the process of carbon emissions reduction in the cruel competitive environment. In contrast, state-owned enterprises undertake more social responsibilities and are subject to government regulations, which may weaken their motivation to pursue financial benefits because of catering to policies. Based on this, two hypotheses are proposed:

*H3*: Technological innovation and carbon performance moderate each other to positively promote corporate financial performance.

*H4*: Compared with state-owned enterprises, the synergy effect of non-state-owned enterprises is more significant.

## Research design and method

### Sample selection and data sources

Using the Chinese A-share energy industry in 2014–2019 as the research sample, this paper examines the impact of carbon performance and technological innovation on corporate financial performance in the energy industry, as well as the synergistic moderating effect of the two. In this study, the sample is processed as follows: (1) excluded listed companies marked as ST and *ST; (2) excluded the companies whose relevant financial data are not available. After the above screening, a total of 1,143 observations in 6 years were finally obtained. The carbon performance data used in this paper are from China Statistical Yearbook and Carbon Emissions Trading Network, and other related data are from China Stock Market & Accounting Research Database (CSMAR) and the CNINFO. The collected sample data were sorted by using EXCEL 2019, and the statistical analysis and test were carried out by using Stata15.0 software.

### Variable design and measurement

#### Dependent variables: Corporate financial performance

There are many measures of corporate financial performance. This paper adopts the Tobin-q, a commonly used indicator in empirical research, as a substitute variable for corporate financial performance. Taking the robustness of empirical studies into further consideration, ROA is finally used as a substitute variable for corporate financial performance.

#### Independent variables: Corporate carbon performance, technological innovation

The so-called carbon performance simply refers to the efforts made by enterprises in carbon emissions. Previous studies have measured carbon performance in terms of carbon emissions, energy consumption, and the degree of development of low-carbon technologieies. Reviewing these studies, it is found that the criterion of carbon emissions is more reliable and scientific. Therefore, this paper refers to Clarkson et al. (2008), using the reciprocal of total carbon emissions per million yuan of net sales as a measure of carbon performance. On this basis, we use the reference value of 0.68/kg of standard coal from the Energy Economics Research Institute of Japan as the conversion factor of carbon emissions. At the same time, in order to examine the persistence of carbon performance and technological innovation on financial performance and to weaken the endogeneity of the reverse causality generated by financial performance with carbon performance and technological innovation, we tested the variables with a lag of one period.

As this paper focuses on the impact of investment in technological innovation, the relative indicator of the ratio of corporate R&D investment to operating revenue is used as a proxy variable for corporate technological innovation, reflecting the efforts made by companies in technological innovation.

#### Control variables

Based on previous research, we controls the impact of company scale, asset-liability ratio, operating income growth rate, separation of two positions, independent director ratio, and cash holdings on corporate financial performance, as well as annual variables. The specific measurement indicators are shown in Table 1.

**Table 1:** Variable definitions.

Nature of variables	Names of variables	Symbols of variables	Definitions
Dependent variables	Corporate financial performance	*Tobin-q*	Market value/Total assets of the company
		*ROA*	Expressed as return on assets
Independent variables	Carbon performance	*CP*	Reciprocal of total carbon emissions per million yuan of net sales
	Technological innovation	*R* & *D*	R & D investment to operating income ratio
Control variables	Asset-liability ratio	*Lev*	Average balance of liabilities/average balance of assets
	Company size	*Size*	Natural logarithm of total business assets
	Operating income growth rate	*Growth*	(Increase in operating income this year/total operating income at the end of last year) × 100%
	CEO duality	*Dua*	Whether the chairman and general manager have two positions in one, 1 for yes, 0 for otherwise
	Independent director ratio	*Bind*	Proportion of independent directors on the board
	Cash holdings	*CF*	Corporate monetary capital/total assets
	Annual dummy variable	*Year*	Year of control

### Model building

In order to examine the impact of carbon performance and technological innovation on corporate financial performance, the following models are established:


$$Tobin-q=\alpha_{1}+\alpha_{2}CP+ControlVariables+\varepsilon \;\left(1\right)$$

$$Tobin-q=\beta_{1}+\beta_{2}R\&D+ControlVariables+\varepsilon \;\left(2\right)$$

$$\begin{array}{c} Tobin-q=\gamma_{1}+\gamma_{2}CP+\gamma_{3}R\&D+\gamma 4CP*R\&D\\ +ControlVariables+\varepsilon \;\left(3\right) \end{array}$$

Eqs. (1) and (2) are, respectively, used to test the impact of carbon performance and technological innovation on corporate financial performance. Eq. (3) investigates the impact of the interaction term between carbon performance and technological innovation on financial performance.

Where Tobin-q represents corporate financial performance, CP represents carbon performance, R&D represents technological innovation, CP*R&D represents the interaction term between carbon performance and technological innovation. ControalVariables represents the interaction term between carbon performance and technological innovation, including asset-liability ratio, company size, operating income growth rate, CEO duality, independent director ratio, cash holdings, annual dummy variable. Constant is denoted by $\beta_{0}$ , while the symbol ε denotes the error term.

## Empirical results

### Descriptive statistics

Table 2 shows the descriptive statistics of the main variables. Preliminarily, the mean value of the explained variable Tobin-q is 2.143 with a standard deviation of 1.521. The mean value of the carbon performance CP is 222.4, which indicates that each ton of CO2 emissions can generate 22.24 million Yuan in sales revenue, and the dispersion between the extreme values also indicates that the level of carbon performance varies greatly between energy industries. The lack of clear industry standards leads to problems such as low energy utilization rate and obvious gap among enterprises, and the emphasis of enterprises on carbon performance is also uneven. The mean value of the technological innovation level of enterprises is 2.548%, which is a small investment in innovation for the energy industry as a whole, which needs technological innovation to maintain competitiveness. Standard deviation 2, indicating obvious differences in technical capital investment. In terms of the control variables, the mean value of corporate asset-liability ratio is 0.458, the mean value of natural logarithm of total assets is 22.740, the mean value of profitability is 0.245, the mean value of the percentage of sole directors is 36.88%, and the mean value of corporate cash holdings is 0.135.

**Table 2:** Descriptive statistics.

Variables	Observations	Mean values	Standard deviations	Minimum values	Maximum values
*Tobin-q*	1,143	2.143	1.521	0.712	14.89
*ROA*	1,143	0.040	0.077	−1.125	0.331
*CP*	1,143	222.4	57.870	64.740	627.1
*R&D*	1,143	2.548	2.007	0.003	11.760
*Lev*	1,143	0.458	0.214	0.055	2.290
*Size*	1,143	22.740	1.403	19.980	26.750
*Growth*	1,143	0.245	2.860	−0.814	96.020
*Dua*	1,143	0.220	0.414	0	1
*Bind*	1,143	36.880	5.111	23.080	62.5
*CF*	1,143	0.135	0.0938	0.005	0.814

### Correlation analysis

In order to avoid the multicollinearity problem between the respective variables and provide more reliable conclusions for the research hypothesis, Pearson correlation analysis is conducted for each variable before the regression, and according to the results in Table 3, the correlation coefficient among the variables is basically lower than 0.5, which indicates that this study is less affected by multicollinearity. And it can be tentatively determined that the carbon performance of the energy industry has a positive relationship with technological innovation and corporate financial performance.

**Table 3:** Correlation analysis.

variables	*Tobin-q*	*CP*	*R* & *D*	*Lev*	*Size*	*Growth*	*Dua*	*Bind*	*CF*
*Tobin-q*	1								
*CP*	0.025	1							
*R&D*	0.380***	−0.194***	1						
*Lev*	−0.411***	−0.017	−0.467***	1					
*Size*	−0.560***	0.235***	−0.478***	0.583***	1				
*Growth*	0.073**	0.137***	−0.023	−0.023	−0.017	1			
*Dua*	0.040	0.001	−0.031	−0.019	0.012	0.028	1		
*Bind*	0.105***	−0.065**	0.166***	−0.163***	−0.214***	0.056*	0.023	1	
*CF*	0.273***	0.003	0.196***	−0.319***	−0.305***	0.013	−0.000	0.131***	1

*, **, *** indicate significant at the 10, 5, and 1% levels, respectively.

### Regression results

Panel data is used to performed multivariate regression analysis, and the interaction term of independent variables is added to the model, and the coefficients of each variable are analyzed to test the existence of the effect. When testing the moderating effect, if the regression coefficient of the interaction term on the dependent variable is significant, it indicates that the moderating variable has a significant moderating effect. The specific empirical results are presented in Table 4:

**Table 4:** The regression results of carbon performance and technological innovation on financial performance.

Variables	Model 1	Model 2	Model 3	Model 1	Model 2	Model 3	*Tobin-q*	*Tobin-q*	*Tobin-q*	*Tobin-q*	*Tobin-q*	*Tobin-q*
*CP*	0.003***		0.005***			
	(5.55)		(7.24)			
*R*&*D*		0.100***	0.121***			
		(5.92)	(7.23)			
CP * R&*D*			0.001***			
			(3.52)			
*L.CP*				0.004***		0.006***
				(5.35)		(6.85)
*L.R*&*D*					0.091***	0.101***
					(4.60)	(5.17)
L.CP * L.R& *D*						0.001***
						(3.58)
*Lev*	−0.701***	−0.629***	−0.331*	−0.555***	−0.551***	−0.272
	(−4.13)	(−3.66)	(−1.92)	(−2.87)	(−2.82)	(−1.39)
*Size*	−0.484***	−0.391***	−0.444***	−0.524***	−0.426***	−0.480***
	(−17.57)	(−14.41)	(−16.12)	(−16.51)	(−13.42)	(−14.90)
*Growth*	0.016	0.027***	0.020**	0.196***	0.168***	0.171***
	(1.61)	(2.73)	(2.07)	(3.22)	(2.73)	(2.86)
*bind*	0.016***	0.017***	0.018***	0.016**	0.017***	0.017***
	(2.81)	(3.06)	(3.26)	(2.48)	(2.69)	(2.75)
*Two*	−0.049	−0.094	−0.076	−0.077	−0.117	−0.107
	(−0.68)	(−1.29)	(−1.07)	(−0.93)	(−1.40)	(−1.30)
*CF*	1.096***	1.133***	0.882**	0.910**	1.024**	0.705*
	(3.12)	(3.23)	(2.54)	(2.28)	(2.55)	(1.77)
*Constant*	11.887***	10.171***	9.304***	13.485***	11.821***	10.981***
	(19.69)	(15.78)	(14.72)	(19.34)	(15.61)	(14.79)
*Year*	YES	YES	YES	YES	YES	YES
*Adj- R^2^*	0.455	0.457	0.482	0.455	0.451	0.477
*F*	78.99	79.63	75.68	71.42	70.20	66.11

*, **, *** indicate significant at the 10, 5, and 1% levels, respectively.

Regressions are conducted on Model 1 and the results are shown in Table 4. After controlling the relevant variables, After controlling for the relevant variables, the coefficient of the explanatory variable (CP) is significantly positive and significant at the 1% level, that is a unit increase in CP leads to an decrease of 0.001 score change in Tobin-q, and the results indicate that carbon performance in the energy sector can significantly improve corporate financial performance. The results are consistent with the findings of He et al. (2017), so Hypothesis 1 is supported. Although the conventional concept holds that it costs a lot more for companies to fulfill their responsibilities related to carbon emissions reduction, the above findings conclude that the benefits of carbon emissions reduction by energy companies are greater than the costs they consume, which can promote the development of companies overall, and this explains why companies are willing to take the initiative to carry out carbon emissions reduction activities. In addition, the improvement of carbon performance of enterprises is an expression of actively fulfilling social responsibilities and sending a signal of green and sustainable development. According to the signal transmission theory, this practice can attract more potential investors, thus improving financial performance and forming a virtuous circle. And with the gradual development of the carbon trading market, the efforts made by enterprises in carbon emissions will be directly realized in the carbon emissions trading market.

The regression of Model 2, the results of which are shown in Table 4, tests the role of technological innovation on the financial performance of firms. The coefficient of enterprise technological innovation (R&D) is significantly positive and significant at the 1% level, that is a unit increase in R&D reduces Tobin-q by 0.1 score, which indicates that the investment in technological innovation in the energy industry can significantly improve corporate financial performance, so Hypothesis 2 is supported. The results are consistent with the majority of scholars’ conclusions that investment in technological innovation is an important guarantee for enterprises to gain growth and sustainable competitiveness, especially for those energy-consuming industries, where reducing energy consumption and improving energy efficiency are the main ways to develop.

On the basis of model 1 and model 2, the interactive phase CP*R&D is added to test. Through the test of Model 3, the coefficient of interaction term is significantly positive and significant at the 1% level, that is a unit percentage increase in CP*R&D increases Tobin-q by 0.001 score. The results show that: on the one hand, technology innovation can significantly positive to adjust the action of carbon performance on financial performance. On the other hand, carbon performance also positively moderates the effect of technological innovation on financial performance, so hypothesis 3 is supported. Energy companies link their carbon emissions reduction activities with technological innovation to provide a forward-looking direction for technological innovation, while technological innovation, as the focus of their carbon emissions reduction activities, demonstrates to the outside world that they are implementing an economically meaningful carbon strategy and that their economic development is sustainable.

Based on the above three models, a lagged one-period regression is conducted, and the results are still significant, indicating the existence of less endogeneity problems to a certain extent. Meanwhile, corporate carbon performance, technological innovation and their interaction terms are positive and significant at the 1% level, indicating that enterprises’ carbon emissions reduction activities and technological innovation investment not only promote their financial performance in the current period, but also have a certain degree of sustainability.

### Further analysis

Through further distinguishing the different nature of property rights, we examine the impact of carbon performance and technological innovation on financial performance in state-owned and non-state-owned enterprises. As shown in Table 5, the regression results indicate that: (1) carbon performance is significantly positive and has a significant enhancing effect in both state-owned and non-state-owned samples. (2) The investment in technological innovation is significantly positive in the sample of non-state-owned enterprises but not in that of state-owned enterprises, indicating that the technological innovation of non-state-owned enterprises is more profit-seeking, and their technological innovation is often profit-oriented and continuously captures value. In contrast, technological innovation by state-owned enterprises may be required to meet social responsibility and may not necessarily generate financial benefits. At the same time, state-owned enterprises, as legal persons under the leadership of the state, are protected by state-owned capital and have little competition pressure, so it is difficult to significantly improve their financial performance in a short time.(3) In the non-state-owned sample, the interaction term of the two is significantly positive, and the synergistic effect of technological innovation and carbon performance can be well exerted, but no significant improvement is found in state-owned enterprises. Hypothesis 4 is supported.

**Table 5:** Regression based on property rights.

Names of variables	State-owned firm sample			Non-state-owned firm sample			Model 1	Model 2	Model 3	Model 1	Model 2	Model 3	*Tobin-q*	*Tobin-q*	*Tobin-q*	*Tobin-q*	*Tobin-q*	*Tobin-q*
*CP*	0.001***		0.003***	0.004***		0.006***
	(2.72)		(2.67)	(4.20)		(5.14)
*R*&*D*		0.017	0.044**		0.123***	0.128***
		(0.93)	(2.20)		(5.03)	(5.35)
CP * R&*D*			0.001			0.001***
			(1.26)			(2.60)
*Lev*	−0.507***	−0.565***	−0.389**	−0.455	−0.404	−0.075
	(−3.53)	(−3.87)	(−2.53)	(−1.44)	(−1.29)	(−0.24)
*Size*	−0.275***	−0.248***	−0.271***	−0.730***	−0.603***	−0.667***
	(−11.54)	(−10.70)	(−11.32)	(−13.15)	(−10.78)	(−11.87)
*Growth*	0.029	0.024	0.020	0.008	0.024**	0.017
	(0.76)	(0.61)	(0.53)	(0.65)	(2.12)	(1.41)
*Bind*	−0.009*	−0.011**	−0.008	0.019**	0.027***	0.024***
	(−1.69)	(−2.07)	(−1.51)	(2.12)	(3.04)	(2.74)
*Dua*	0.036	0.036	0.041	−0.087	−0.153	−0.120
	(0.36)	(0.36)	(0.42)	(−0.91)	(−1.60)	(−1.28)
*CF*	1,516***	1,251***	1,571***	0.722	1,261***	0.528
	(3.77)	(3.19)	(3.90)	(1.43)	(2.60)	(1.06)
*Constant*	8,228***	7,995***	6,199***	16.675***	14.181***	13.763***
	(15.70)	(14.33)	(11.35)	(13.49)	(10.88)	(10.66)
*Year*	YES	YES	YES	YES	YES	YES
*Adj- R^2^*	0.435	0.428	0.439	0.456	0.462	0.483
*F*	33.14	32.17	28.97	44.33	45.50	42.49

*, **, *** indicate significant at the 10, 5, and 1% levels, respectively.

### Robustness test result

The robustness test still takes the listed enterprises in the energy industry from 2014 to 2019 as a sample. The method of substitution variable is used to test, and ROA is used as the substitution variable of corporate financial performance. At the same time, the lagged one-period test of carbon performance and technological innovation is also conducted. The robustness test remains significant that carbon performance and technological innovation have a positive impact on corporate financial performance, the carbon performance has a significant impact at the 1% level and the regression coefficient is positive; the technological innovation has a significant impact at the 10% level and the regression coefficient is positive; the interaction term has a significant impact at the 1% level and the regression coefficient is positive. The carbon performance and technological innovation have a positive impact on the financial performance of enterprises and their effects are not limited to the current period, but act in the far future to continue the value growth of the company. In summary, the results of the robustness test are basically consistent with the regression tests. The regression results are shown in Table 6.

**Table 6:** Robustness test results.

Names of variables	Model 1	Model 2	Model 3	Model 1	Model 2	Model 3	*ROA*	*ROA*	*ROA*	*ROA*	*ROA*	*ROA*
*CP*	0.000***		0.001***			
	(10.19)		(11.52)			
*R*&*D*		0.003**	0.005***			
		(2.12)	(4.17)			
CP * R&*D*			0.000***			
			(5.38)			
*L.CP*				0.000***		0.000***
				(6.03)		(6.62)
*L.R&D*					0.003**	0.003**
					(2.00)	(2.54)
L.CP * L.R&*D*						0.000***
						(2.84)
Control variables	YES	YES	YES	YES	YES	YES
*Adj-R ^2^*	0.293	0.232	0.318	0.276	0.251	0.285
*F*	40.52	29.68	39.08	33.74	29.78	30.09

## Conclusion and recommendations

### Conclusion

In this article, we take China’s A-share energy industry from 2014 to 2019 as an example to study the relations among carbon performance,technological innovation and corporate financial performance. The results show that carbon performance can positively affect financial performance, which means that the better of carbon performance will promote the better of financial performance. This outcome of the study is in line with the prior work of He et al. (2017), Gaigné et al. (2020), Zhu and Zhang (2022), Lu et al. (2021), Li and Lin (2021), Yuan and Guangpei (2021), and technological innovation can also positively affect financial performance significantly. In other words, both carbon performance and technological innovation are driving factors of corporate financial performance. The finding of the impact of technological innovation on financial performance is consistent with most researchers as discussed in the literature review (Yeguang and Bo, 2018; Tariq et al., 2019; Yuan and Guangpei, 2021; Lei et al., 2022). But only few studies focus on empirical research on the relation between carbon performance and financial performance. Although a lot of researchers have been doing many researches on the impact of CSR on financial performance (Lu et al., 2014; Akben-Selcuk, 2019; Ali et al., 2020). CSR performance is multidimensional or multi-faceted, and carbon emission is just one part of CSR performance. The previous studies of the impact of CSR on financial performance cannot well reflect the value of developing a low-carbon economy for enterprises’ sustainable development. Our finding can provide the direct evidence that carbon performance is also one of the important driving factors of financial performance.

According to regression results, we also found that carbon performance and technological innovation have synergistic effect on firm’s financial performance, which means that technology innovation plays moderating role between carbon performance and financial performance, and carbon performance also plays moderating role between technology innovation and financial performance. Most Previous studies have shown that technology innovation is effective strategy to enterprises’ sustainable development and seldom show that self-imposed emissions reduction is also important to maximize profit. Most researchers directly or indirectly studied the impact of carbon performance and technological innovation on financial performance separately. The innovation of our article lies in the combination of the two. Our finding of coupling synergy effect of carbon performance and technological innovation on financial performance, verify that the interaction of carbon performance and technological innovation is much more effective to corporate financial performance promotion. Technological innovation combined with positive carbon emissions reduction activities are the most effective countermeasure to promote low-carbon transformation and sustainable development of China’s energy enterprises under China’s “dual carbon” background.

In addition, according to group test of different property rights of enterprises, enterprises are divided into state-owned enterprises and non-state-owned enterprises for grouping test, the results show that the carbon performance has a significant enhancement effect in both of them, and the technological innovation input is significantly positive in non-state-owned enterprises but not significant in state-owned enterprises. The possible reason is that the technological innovation of non-state-owned enterprises is more profit-driven, and the technological innovation of state-owned enterprises might be to meet the needs of more else social responsibility, but it does not necessarily produce financial benefits. we still found that the synergistic effects of carbon performance and technological innovation on corporate financial performance is positively significant in non-state-owned enterprises, but not in state-owned enterprises. The Coupling synergy effect of the two is only significant in non-state-owned enterprises., from which we can conclude non-state-owned enterprises have been using both low carbon emission and technological innovation to drive financial performance. China’s non-state-owned enterprises have been taking advantage of the positive effect of carbon emissions reduction on financial performance under the current environmental policy due to capital profit-seeking nature. The above results provide effective strategy for the realization of China’s dual carbon goal a.

At present, China’s carbon emissions reached 12.849 billion tons, has become the world’s highest carbon emissions country. China’s energy sector accounts for more than 80% of China’s total carbon emissions and is the main battleground for achieving the dual carbon target. Under the national “dual carbon” goal, low-carbon transformation and green development of energy industry will play an important role in reaching carbon emission reduction goal. We propose that the multiple-application of technological innovation and self-imposed emissions reduction is the most effective strategy for China’s energy industry sustainable development under China’s “dual carbon”background.

### Recommendations

Based on the above research, this paper puts forward the following recommendations: Firstly, in order to achieve the “dual carbon” goal proposed by China as scheduled, the low-carbon transformation of the energy industry is particularly important, and the government needs to increase the guidance of carbon emissions reduction activities in the energy industry, such as providing technical guidelines for pollution reduction and carbon reduction, and providing incentives for enterprises to carry out carbon emissions reduction activities. In addition, the government needs to formulate specific carbon performance evaluation system and industry standards, train relevant leaders of enterprises, pass on the concept of energy conservation and emission reduction, and teach carbon performance calculation methods, so as to create a good competitive environment for the whole society to pursue green development. At the same time, it is necessary to improve the trading of the carbon market, so that enterprises can see the benefits of carbon emissions reduction and truly integrate carbon reduction activities into their development. Secondly, the reform of state-owned enterprise system has reached a critical stage, where it is necessary to change the conventional development concept of state-owned enterprises, to carry out strategic innovation and institutional innovation, such as selecting executives with social responsibility awareness. At the same time, according to the high-level echelon theory, combined with background characteristics, enrich the talent selection mechanism, improve the management structure. In this way, these state-owned enterprises can adapt to the current low-carbon economic development situation, playing a good role of state-owned assets, and improving the economic efficiency of. Thirdly, enterprises need to change the conventional concept of carbon emissions reduction activities. Enterprises’ investment to reduce carbon emissions is not just a blind cost. The reduction of energy consumption, the improvement of efficiency and the attention of investors will bring actual benefits to the enterprise. Integrating carbon emissions reduction activities into the process of formulating development strategies of enterprises and implementing effective carbon strategies can form a special competitive advantage. For enterprises, especially for energy enterprises, which can use digital energy cost analysis to improve the practicality of technological innovation. Fourthly, enterprises should choose the direction of technological innovation properly, set up an assessment system for “responsible innovation investment” for the innovation department, strictly supervise the flow of technical capital input, and regularly conduct results inspection, and make good use of the innovation resources to make the technological innovation results unique and forward-looking.

One limitation of this paper lies in the measuring of carbon performance. We did not compare the effectiveness of current different measuring methods. Carbon performance should be a comprehensive evaluation method, which can be constructed to evaluate enterprise carbon performance more scientifically. In our article, it might not be persuasive enough to use the inverse of total carbon emissions per million yuan of net sales as a measure of carbon performance, which can be further considered in our future research.

## Data availability statement

The original contributions presented in the study are included in the article/supplementary material, further inquiries can be directed to the corresponding author.

## Author contributions

JY has contributed to the definition of research objectives, developing models, hypotheses, data analysis, and writing original draft. HZ, JM, and YZ has contributed to data collection, develop hypotheses, article writing, revision, and editing. AR has contributed to methodology, validation of results, hypothesis revision, and article revision. All authors contributed to the article and approved the submitted version.

## Conflict of interest

The authors declare that the research was conducted in the absence of any commercial or financial relationships that could be construed as a potential conflict of interest.

## Publisher’s note

All claims expressed in this article are solely those of the authors and do not necessarily represent those of their affiliated organizations, or those of the publisher, the editors and the reviewers. Any product that may be evaluated in this article, or claim that may be made by its manufacturer, is not guaranteed or endorsed by the publisher.
